# Prediagnostic blood biomarkers for pancreatic cancer: meta-analysis

**DOI:** 10.1093/bjsopen/zrae046

**Published:** 2024-06-27

**Authors:** Axel Bengtsson, Tomasz Draus, Roland Andersson, Daniel Ansari

**Affiliations:** Department of Surgery, Clinical Sciences Lund, Lund University, Skåne University Hospital, Lund, Sweden; Department of Surgery, Clinical Sciences Lund, Lund University, Skåne University Hospital, Lund, Sweden; Department of Surgery, Clinical Sciences Lund, Lund University, Skåne University Hospital, Lund, Sweden; Department of Surgery, Clinical Sciences Lund, Lund University, Skåne University Hospital, Lund, Sweden

## Introduction

Pancreatic cancer is often detected at a late stage and usually leads to significant morbidity and deaths^1^. The 5-year survival rate for those with pancreatic cancer detected at a resectable stage is 25%, compared with a 5-year survival rate of 3% for those with pancreatic cancer that is not resectable^2–4^. Some 20% of patients have resectable disease at the time of diagnosis, underscoring the necessity to develop novel early detection tools^2^.

The timeline for the development of pancreatic cancer is controversial. The evolution of pancreatic cancer is probably a slow process, involving sequential, independent accumulation of mutations in both oncogenes and tumour suppressor genes, with progression from precancerous stages to increasingly invasive and metastatic stages over many years^5^. An alternative evolution might be driven by punctuated equilibrium, a process that encompasses intervals of stability and rapid transformation with molecular changes^6^. Epidemiological data suggest that, once clinically apparent, pancreatic cancer may progress from category T1 to T4 in 1 year^7^. Methods must be developed to detect the disease before the onset of clinical symptoms.

Liquid biopsy approaches have garnered substantial interest for pancreatic cancer screening, either as single-cancer tests or as part of multi-cancer early detection tests. A wide range of blood-based biomarkers have been investigated, including proteins, metabolites, microRNAs (miRs), circulating tumour DNA, circulating tumour cells, and exosomes^8^. Previous studies have focused on biomarkers at the time of clinical diagnosis. It remains unknown whether such biomarkers could be used for longitudinal screening in asymptomatic individuals.

The aim of this meta-analysis was to evaluate the use of blood-based biomarkers for the detection of pancreatic cancer in prediagnostic settings, with a particular focus on diagnostic accuracy at different time points up to 5 years before clinical diagnosis.

## Methods

The authors conducted a systematic review in accordance with the PRISMA guidelines^9^. The review was pre-registered in PROSPERO, the international prospective register of systematic reviews (CRD42023444953). PubMed, Embase, the Cochrane Library, and the Web of Science were searched from 1 January 2000 to 30 June 2023. Human studies assessing the prediagnostic performance of circulatory biomarkers for the detection of pancreatic cancer were included. Publications providing area under the receiver operating characteristic (ROC) curve (AUC) values for up to 5 years before diagnosis were included. A meta-analysis was performed by calculating the weighted summary AUC under the fixed-effects model. Details regarding the full methods, statistical analysis, and risk-of-bias assessment can be found in the *Supplementary material*.

## Results

The literature search produced 906 records, of which 122 potentially eligible studies were selected for full-text evaluation. After evaluation of eligibility, 12 studies^10–21^ were included (*Fig. S1*). A summary of the included studies is given in *Table 1*.

**Table 1:**
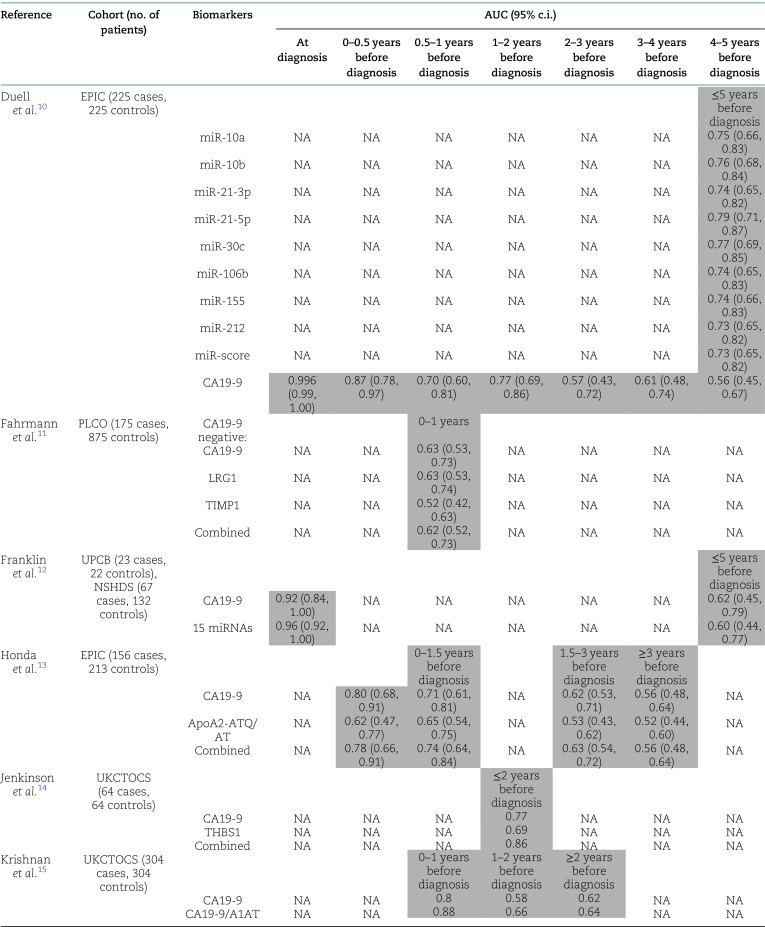

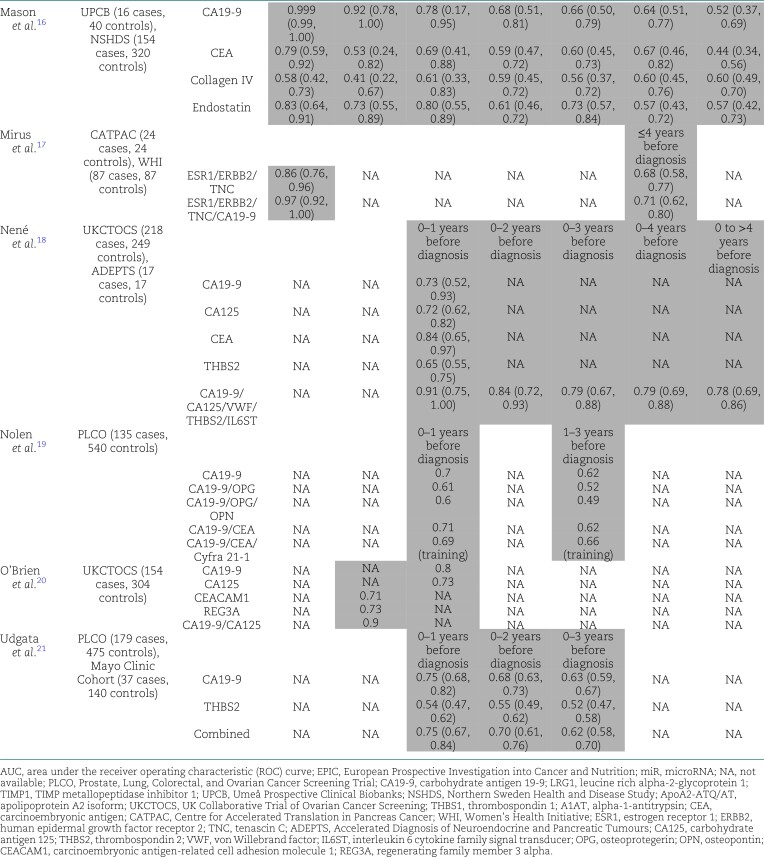
Study characteristics

### Carbohydrate antigen 19-9

**Fig. 1:**
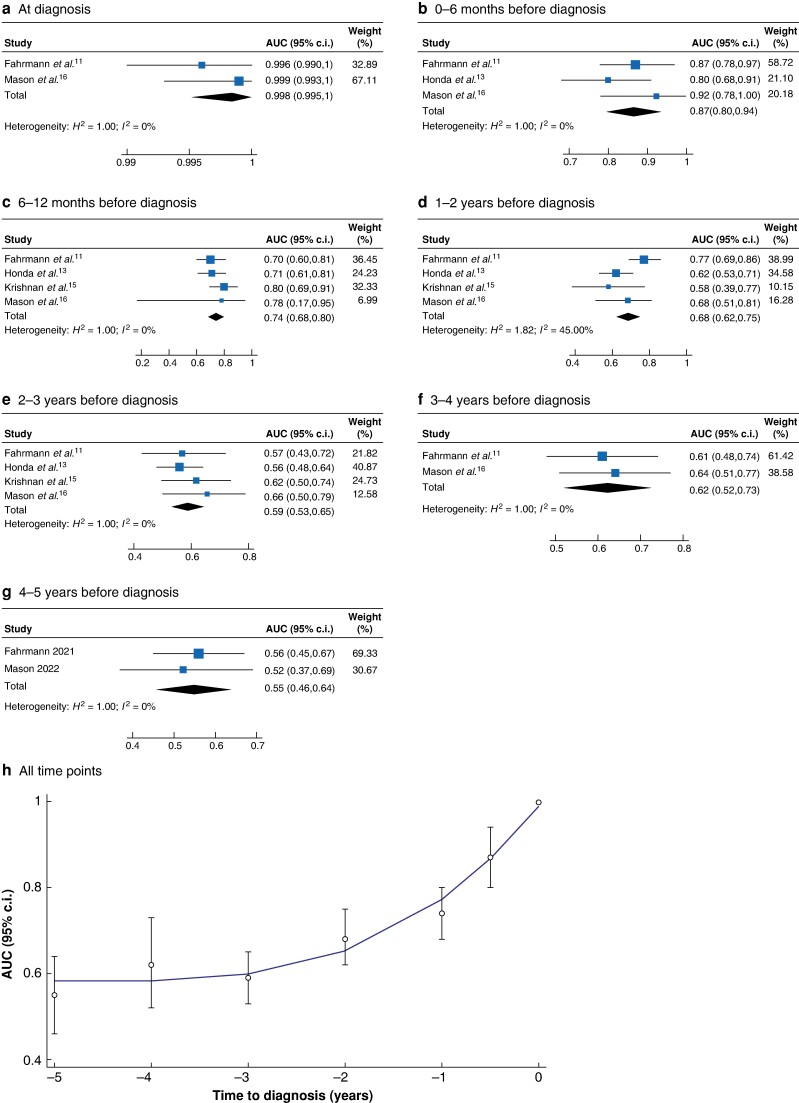
Carbohydrate antigen 19-9 performance in relation to time to diagnosis of pancreatic cancer **a–g** Forest plots at different time intervals. **h** Scatter plot with overlaid fractional-polynomial prediction plot for all time points.

A total of 11 studies^11–21^ investigated the prediagnostic accuracy of carbohydrate antigen 19-9 (CA19-9) for the detection of pancreatic cancer. After excluding studies with overlapping populations^18–21^, four studies^11,13,15,16^ based on five different cohorts (Prostate, Lung, Colorectal, and Ovarian Cancer Screening Trial (PLCO), European Prospective Investigation into Cancer and Nutrition (EPIC), UK Collaborative Trial of Ovarian Cancer Screening (UKCTOCS), Umeå Prospective Clinical Biobanks (UPCB), and Northern Sweden Health and Disease Study (NSHDS)) were available for meta-analysis. The forest plots are shown in *Fig. 1a–g*. The AUC was excellent (0.998) at diagnosis, good (0.87) at 6 months before diagnosis, and fair (0.74) at 12 months before diagnosis, but poor (0.55) at 5 years before diagnosis (*Fig. 1h*).

### Protein biomarkers

Several studies explored the prediagnostic capabilities of alpha-1-antitrypsin (A1AT)^15^, apolipoprotein A2 (ApoA2) isoform^13^, carbohydrate antigen 125 (CA125)^18,20^, carcinoembryonic antigen (CEA)^16,18,19^, carcinoembryonic antigen-related cell adhesion molecule 1 (CEACAM1)^20^, collagen IV^16^, endostatin^16^, osteoprotegerin (OPG)^19^, osteopontin (OPN)^19^, regenerating family member 3 alpha (REG3A)^20^, thrombospondin 1 (THBS1)^14^, and thrombospondin 2 (THBS2)^18,21^ for the detection of pancreatic cancer. Most of these markers had limited discriminatory power and provided no major improvement over CA19-9 alone, as shown in *Table 1*. A three-marker panel (comprising estrogen receptor 1 (ESR1), human epidermal growth factor receptor 2 (ERBB2), and tenascin C (TNC)) was able to detect pancreatic cancer with an AUC of 0.86 at diagnosis and an AUC of 0.68 within 4 years of diagnosis^17^. Adding CA19-9 to the panel improved the discriminatory power at diagnosis (AUC = 0.97) and for the prediagnostic samples (AUC = 0.71). In another study, a five-marker panel (comprising CA19-9, CA125, von Willebrand factor (VWF), THBS2, and interleukin 6 cytokine family signal transducer (IL6ST)) detected pancreatic cancer with an AUC of 0.91 within 1 year of diagnosis, which was superior to CA19-9 alone (AUC = 0.73). The five-marker panel had an AUC of 0.78 up to 4 years before diagnosis^18^.

### microRNAs

There were two studies^10,12^ that evaluated the prediagnostic utility of miRs for the detection of pancreatic cancer. Of these studies, one study^10^ reported on the discriminatory power of eight different miRs and found that the AUC was highest for miR-21-5p (0.79) within 5 years of diagnosis. Another study^12^ reported that a panel of 15 miRs can detect pancreatic cancer with an AUC of 0.96 at the time of diagnosis, but the performance dropped to 0.60 within 5 years.

## Discussion

This was the first meta-analysis on the accuracy of prediagnostic biomarkers for the detection of pancreatic cancer. Several established biomarker candidates derived from diagnostic research delivered below-par performance for prediagnostic samples. Only CA19-9 had an acceptable performance within 1 year of diagnosis, but the discriminatory power diminished at extended time points. These findings suggest that CA19-9 is a reliable biomarker for pancreatic cancer, but there is a need for additional markers. None of the biomarker combinations tested had clinical superiority compared to CA19-9 alone.

A limitation of this meta-analysis was the heterogeneity in designs, patient cohorts, and analytical techniques among studies, which may limit the generalization of these findings. Publication bias cannot be excluded, as positive biomarker results are more likely to be published, potentially leading to an overestimation of the true effect size.

In the future, more research should be directed towards the identification of biomarkers for pancreatic cancer that predate the current time of clinical detection. Analysing prediagnostic samples from individuals who later develop pancreatic cancer can help identify biomarkers associated with disease onset and early-stage disease. Relevant prediagnostic samples can be collected via prospective cohort studies, RCTs, or surveillance programmes of high-risk individuals (for example those with a family history/genetic susceptibility, new-onset diabetes, or cystic lesions). A multiplex biomarker panel integrating multiple promising biomarkers into a single assay offers a comprehensive and efficient approach to increase diagnostic accuracy. Machine-learning algorithms are well suited for diagnostic classification problems, especially when using large data sets^22^.

## Supplementary Material

zrae046_Supplementary_Data

## Data Availability

Data supporting this study are included within the article and *Supplementary material*.
