# Supplementary material for: Prediagnostic blood biomarkers for pancreatic cancer: meta-analysis
Source: BJS Open. 2024 Jun 27;8(3):zrae046. doi: 10.1093/bjsopen/zrae046 (PMC11210304; doi:10.1093/bjsopen/zrae046)
Supplement: zrae046_Supplementary_Data [file zrae046_supplementary_data.docx]

**Pre-diagnostic Blood Biomarkers for Pancreatic Cancer: Meta-analysis**

Axel Bengtsson, Tomasz Draus, Roland Andersson, Daniel Ansari

Department of Surgery, Clinical Sciences Lund, Lund University, Skåne University Hospital, Lund, Sweden

**Corresponding author:**

Daniel Ansari, MD, PhD, Associate Professor

Department of Surgery, Clinical Sciences Lund

Lund University, Skåne University Hospital, Lund

SE-221 85 Lund, Sweden

Tel: + 46 46 222 46 72

E-mail: [daniel.ansari@med.lu.se](mailto:daniel.ansari@med.lu.se)

ORCID ID: 0000-0002-4357-822X

**Supplementary Materials - Index**

| **Supplementary Methods** |  |
| --- | --- |
| Search strategy | *page 2* |
| Eligibility criteria | *page 2* |
| Screening | *page 3* |
| Quality assessment and risk of bias | *page 3* |
| Data processing and interpretation | *page 3* |
| **Supplementary Results** |  |
| PRISMA diagram | *page 4* |
| Risk of bias and applicability  Sensitivity analyses  Publication bias | *page 4*  *page 5*  *page 5* |
| **References** | *page 6* |
|  |  |

**Supplementary Methods**

A summary of the search strategy is shown in Table S1.

**Table S1. Search strategy**

| **Database** | **Search terms** |
| --- | --- |
| PubMed | ((prediagnostic) OR (pre-diagnostic)) AND ((pancreas cancer) OR (pancreatic cancer)), and EPIC, Malmö Diet Cancer Study, Malmö Preventive Project, Malmö Offspring Study, Northern Sweden Health Study, PLCO, UKCTOS, Mayo Clinic Biobank, PHS, WHI, WHS, HPFS, NHS, ATBC, BCDDP, CNBSS; CPS-II, CTS, COSM, IWHS, MCCS, NLCS, NYSC, SMC combined with AND (pancreatic cancer)) AND (biomarker). |
| Embase | (prediagnostic OR 'pre-diagnostic') AND ('pancreas cancer'/exp OR 'pancreas cancer'), and EPIC, Malmö Diet Cancer Study, Malmö Preventive Project, Malmö Offspring Study, Northern Sweden Health Study, PLCO, UKCTOS, Mayo Clinic Biobank, PHS, WHI, WHS, HPFS, NHS, ATBC, BCDDP, CNBSS; CPS-II, CTS, COSM, IWHS, MCCS, NLCS, NYSC, SMC combined with AND ('pancreas cancer'/exp OR 'pancreas cancer') AND biomarker. |
| Cochrane Library | prediagnostic in All Text OR pre-diagnostic in Title Abstract Keyword AND pancreatic cancer in Title Abstract Keyword, and EPIC, Malmö Diet Cancer Study, Malmö Preventive Project, Malmö Offspring Study, Northern Sweden Health Study, PLCO, UKCTOS, Mayo Clinic Biobank, PHS, WHI, WHS, HPFS, NHS, ATBC, BCDDP, CNBSS; CPS-II, CTS, COSM, IWHS, MCCS, NLCS, NYSC, SMC combined with “biomarker” in Title Abstract Keyword AND pancreatic cancer in Title Abstract Keyword. |
| Web of Science | ALL=(prediagnostic OR pre-diagnostic) AND ALL=(pancreatic cancer) and, and EPIC, Malmö Diet Cancer Study, Malmö Preventive Project, Malmö Offspring Study, Northern Sweden Health Study, PLCO, UKCTOS, Mayo Clinic Biobank, PHS, WHI, WHS, HPFS, NHS, ATBC, BCDDP, CNBSS; CPS-II, CTS, COSM, IWHS, MCCS, NLCS, NYSC, SMC combined with ALL=(pancreatic cancer) AND ALL=(biomarker) |

**Eligibility criteria**

Human studies were eligible in which blood biomarkers were used to discriminate between pancreatic cancer and control populations in a pre-diagnostic setting. Studies with less than 50 individuals were excluded. Publications had to provide area under the ROC curve (AUC) values 0 to 5 years before diagnosis to be considered for inclusion. Review articles were excluded. An English-language restriction was applied.

**Screening**

Two reviewers (A.B. and T.D.) performed the search and checked the titles and abstracts against the eligibility criteria. Full text studies were then checked against inclusion criteria. Any disagreements were resolved involving a third reviewer (D.A.).

**Quality assessment and risk of bias**

All included studies were assessed using the QUADAS-2 and QUADAS-C tools^1^.

**Data processing and interpretation**

Two reviewers (A.B. and T.D.) independently extracted the data using prespecified forms. A meta-analysis was performed by calculating the weighted summary AUC under the fixed effects model as described by Zhou et al^2^. The fixed effects model is preferred when the number of included studies is less than five^3^. The calculation of pooled AUC requires the standard error of the AUC. When the standard error was not reported in the studies, we used the formula suggested by Hanley and McNeil^4^

$$SE\left( AUC \right)=\sqrt{\frac{AUC\left( 1-AUC \right)+\left( N_{1}-1 \right)\left( Q_{1}-{AUC}^{2} \right)+(N_{2}-1)(Q_{2}-{AUC}^{2})}{N_{1}N_{2}}}$$

where

$$Q_{1}= \frac{AUC}{2-AUC}$$

$$Q_{2}= \frac{{2AUC}^{2}}{1+AUC}$$

Heterogeneity was measured using the I^2^ and H^2^ statistics. All statistical analyses were performed with STATA/MP version 18 (StataCorp LP, College Station, TX, USA) and MedCalc version 22.016 (MedCalc Software, Ostend, Belgium).

**Supplementary Results**

**PRISMA diagram**

The study selection process is shown in Figure S1.****

**Figure S1.** PRISMA diagram for the review

**Risk of bias and applicability**

Assessment of the included studies using the QUADAS-2 and QUADAS-C tools revealed low risk of bias and applicability concerns across most domains (Table S2 and Figure S2).

**Table S2. QUADAS assessment of included studies**

| Study | **Risk of bias**  **(QUADAS-2)** | | | | **Applicability concerns**  **(QUADAS-2)** | | | | | **Risk of bias**  **(QUADAS-C)** | | | |
| --- | --- | --- | --- | --- | --- | --- | --- | --- | --- | --- | --- | --- | --- |
|  | **P** | **I** | **R** | **FT** |  | **P** | **I** | **R** |  | **P** | **I** | **R** | **FT** |
| Duell, 2017 | ✓ | ? | ✓ | ✓ |  | ✓ | ✓ | ✓ |  | ✓ | ✓ | ✓ | ✓ |
| Fahrmann, 2021 | ? | ? | ✓ | ✓ |  | ? | ? | ? |  | ✓ | ? | ✓ | ✓ |
| Franklin, 2018 | ? | ? | ? | ✓ |  | ✓ | ? | ✓ |  | ✓ | ✓ | ✓ | ✓ |
| Honda, 2019 | ✗ | ✗ | ✗ | ? |  | ✓ | ✗ | ? |  | ✗ | ✗ | ? | ✗ |
| Jenkinson, 2016 | ✓ | ✗ | ✓ | ✓ |  | ✓ | ✓ | ✓ |  | ✓ | ? | ✓ | ✓ |
| Krishnan, 2017 | ✓ | ✗ | ✓ | ✓ |  | ✓ | ✓ | ✓ |  | ✓ | ? | ✓ | ✓ |
| Mason, 2022 | ✓ | ✓ | ✓ | ✓ |  | ✓ | ✓ | ✓ |  | ✓ | ✓ | ✓ | ✓ |
| Mirus, 2015 | ✓ | ✗ | ? | ? |  | ✓ | ✓ | ? |  | ✓ | ✓ | ✗ | ✗ |
| Nené, 2023 | ✓ | ✓ | ✓ | ✓ |  | ✓ | ✓ | ✓ |  | ✓ | ✓ | ✓ | ✓ |
| Nolen, 2014 | ✓ | ✓ | ✓ | ✓ |  | ✓ | ✓ | ✓ |  | ✓ | ✓ | ✓ | ✓ |
| O’Brien, 2015 | ✓ | ✓ | ✓ | ✓ |  | ✓ | ✓ | ✓ |  | ✓ | ✓ | ✓ | ✓ |
| Udgata, 2021 | ✓ | ✓ | ✓ | ✓ |  | ✓ | ✓ | ✓ |  | ✓ | ✓ | ✓ | ✓ |

P = patient selection; I = index test; R = reference standard; FT = flow and timing. ✓ indicates low risk; ✗ indicates high risk; ? indicates unclear risk.

**Figure S2.** Risk of bias and applicability concerns

**Sensitivity analyses**

We performed sensitivity analyses, in which the study conducted by Honda et al^5^ was removed due to slightly different time intervals for biomarker evaluation as compared to the other studies. The results indicated that the weighted summary AUCs before and after the removal of the study were similar at large, suggesting solidity of the meta-analysis results. With the study by Honda et al included, the weighted summary AUC was 0.74 (0.68-0.80) at 6-12 months before diagnosis, 0.68 (0.62-0.75) at 1-2 years before diagnosis and 0.59 (0.53-0.65) at 2-3 years before diagnosis, compared to 0.75 (0.68-0.82), 0.72 (0.64-0.80) and 0.61 (0.53-0.69) at these time points, respectively, after exclusion of the study by Honda et al.

**Publication bias**

No formal assessment of publication bias was performed, as traditional methods such as funnel plots and regression tests are not suitable for diagnostic accuracy studies^6^. We used a comprehensive search strategy of multiple databases to capture relevant studies. However, only published data were considered and no gray literature search was conducted and study authors were not contacted to obtain missing data.

**References**

1. Yang B, Mallett S, Takwoingi Y, Davenport CF, Hyde CJ, Whiting PF, et al. QUADAS-C: A Tool for Assessing Risk of Bias in Comparative Diagnostic Accuracy Studies. *Ann Intern Med* 2021;**174**:1592-1599.

2. Zhou X, Obuchowski N, McClish D. *Statistical methods in diagnostic medicine*. New York: Wiley, 2002.

3. Tufanaru C, Munn Z, Stephenson M, Aromataris E. Fixed or random effects meta-analysis? Common methodological issues in systematic reviews of effectiveness. *Int J Evid Based Healthc* 2015;**13**:196-207.

4. Hanley JA, McNeil BJ. The meaning and use of the area under a receiver operating characteristic (ROC) curve. *Radiology* 1982;**143**:29-36.

5. Honda K, Katzke VA, Hüsing A, Okaya S, Shoji H, Onidani K, et al. CA19-9 and apolipoprotein-A2 isoforms as detection markers for pancreatic cancer: a prospective evaluation. *Int J Cancer* 2019;**144**:1877-1887.

6. Macaskill P, Gatsonis C, Deeks JJ, Harbord RM, Takwoingi Y. Chapter 10: Analysing and Presenting Results. In: Deeks JJ, Bossuyt PM, Gatsonis C (editors), Cochrane Handbook for Systematic Reviews of Diagnostic Test Accuracy Version 1.0. The Cochrane Collaboration, 2010. Available from: <http://srdta.cochrane.org/>.
